# Formation of MXene-Derived/NiCoFe-LDH Heterostructures for Supercapacitor Applications

**DOI:** 10.3390/ma16041643

**Published:** 2023-02-16

**Authors:** Yihan Guo, Tongxiang Chen, Yongjin Zou

**Affiliations:** Guangxi Key Laboratory of Information Materials, Guilin University of Electronic Technology, Guilin 541004, China

**Keywords:** supercapacitor, MXene, layered double hydroxide, three-dimensional

## Abstract

In this study, MXene-derived/NiCoFe-LDH heterostructures with three-dimensional interconnected porous network microstructures were prepared, leveraging the excellent electrical conductivity and growth platform provided by the MXene material. The remarkable specific capacitance of metal oxides was fully exploited. The composite exhibited high specific capacitance and excellent stability, with a specific capacitance of 1305 F g^−1^ at 1 A g^−1^ and a capacitance of 85.7% of the initial performance after 6000 charge/discharge tests at 10 A g^−1^. A two-electrode assembly was constructed using activated carbon as the negative electrode material corresponding to 49.5 Wh kg^−1^ at 800 W kg^−1^, indicating that the electrodes could achieve rapid charge/discharge. The findings of this study indicate that the composite material comprising LDH/MXene has significant potential for supercapacitor applications.

## 1. Introduction

With the continuous improvement in social productivity, the demand for fossil fuels is increasing. However, the quantity of fossil fuels is limited because it cannot be regenerated in a short period, and their use results in by-products that are hazardous to the ecosystem [1,2,3]. Therefore, the development of new energy sources is of strategic importance to alleviate the fossil energy crisis. However, new energy sources are limited by natural factors and cannot be used “as is”; hence, it is especially important to find energy storage equipment with high energies and densities [4,5,6]. To solve this problem, a hybrid supercapacitor device based on battery-type and double-layer electrodes, for which the comprehensive performance compensates for the limitations of both batteries and supercapacitors, was constructed. To further optimize its performance, suitable electrode materials must be developed.

Two-dimensional transition metal carbide/nitride (MXene) is a newly discovered two-dimensional material with the formula M_n+1_X_n_T_x_, where M, X, and T represent a transition metal, carbon or nitrogen, and some functional groups (e.g., OH), respectively [7,8,9,10]. Thus, it also has good hydrophilicity and can be used as an ideal electrode material. Simple multilayer Ti_3_C_2_T_x_ electrodes prepared using the lithium fluoride and hydrochloric acid etching methods reported by Lukatskaya et al. were electrochemically tested to obtain an electrode material with a specific capacitance of 380 F g^−1^ [11].

Layered double hydroxides (LDHs) are a representative class of pseudocapacitive materials with high specific capacitance and electrochemical activities that are considerably higher than those of carbon materials [12,13,14,15,16]. Liu et al. obtained a high specific capacitance (1464.7 F g^−1^) using a hydrothermal method to grow NiCo–LDH on nickel foam, which was prepared as an electrode [17]. However, owing to its poor crystallization properties during charge/discharge, this material is prone to decomposition under acidic or alkaline conditions, which changes the morphology of the original material and degrades its performance. The cyclic performance decreases to less than 90% after a few hundred charge/discharge cycles. Therefore, researchers have attempted to improve the cycling performance by compounding with other materials. Cai et al. used a simple one-step hydrothermal method to combine NiCo–LDH and reduced graphene oxide with a negative surface charge, achieving an ultra-high specific capacitance (1911.1 F g^−1^). The electrode material can also exhibit a specific capacitance of 1469.8 F g^−1^ at a high current density of 20 A g^−1^ and better stability than the abovementioned materials after multiple cycles [18].

In this study, we prepared heterostructured NiCoFe–LDH/V_2_CT_x_–MXene composites with interconnected three-dimensional networks by combining V_2_CT_x_–MXene with LDH. The specific capacitance of the electrode was 1305 F g^−1^ at 1 A g^−1^, and asymmetric supercapacitors were fabricated using activated carbon (AC) as the anode material.

## 2. Materials and Methods

### 2.1. Material Synthesis

Ni(NO_3_)_2_·6H_2_O (99%),Co(NO_3_)_2_·6H_2_O (99%), Fe(NO_3_)_3_·9H_2_O (99%), and urea (99%) were purchased from Shanghai Aladdin Reagent Co., Ltd. (Shanghai, China). V_2_AlC was purchased from Jilin Yiyi Technology Co., Ltd. (Jilin, China). HF was purchased from Xilong Chemical Co. (Shantou, China).

#### 2.1.1. Synthesis of V_2_CT_x_–MXene

V_2_AlC–MAX (1 g) was added to a container containing 40% HF solution (50 mL) in ten times and stirred for 95 h at 40 °C, followed by centrifugation at 3500 r min^−1^ for 5 min to leave the lower solid layer. This operation was continued for 7–9 times until the solution appeared neutral. During the last centrifugation, anhydride ethanol was used to wash the precipitate, after which the precipitate was collected and placed in a vacuum oven to obtain V_2_CT_x_–MXene nanosheets.

#### 2.1.2. Synthesis of NiCoFe–LDH/V_2_CT_x_–MXene

Briefly, 1 mM Ni(NO_3_)_2_·6H_2_O, 0.5 mM Co(NO_3_)_2_·6H_2_, 0.5 mM Fe(NO_3_)_3_·9H_2_O, and 0.6 mM urea were dissolved in deionized water (30 mL). Then, 0.7 mM C_6_H_8_O_7_·H_2_O and V_2_CT_x_–MXene (0.1 g) were added to the solution, and it was continuously agitated for 3 h at room temperature. Then, the mixed homogeneous solution was transferred to a PTFE (100 mL) reactor and heated at 120 °C for 10 h. After the reaction was complete, the collected precipitate was rinsed with deionized water six times. The sample was placed in a 60 °C vacuum oven for 12 h to obtain NiCoFe–LDH/V_2_CT_x_–MXene.

### 2.2. Material Characterization

The microscopic morphology and elemental composition of the material were described by field emission scanning electron microscopy (SEM, Quanta FEG 450, FEI, Hillsboro, OR, USA) and field emission transmission electron microscopy (TEM, Talos F200X). The surface elements of the samples were analyzed by electron binding energy using X-ray photoelectron spectroscopy (XPS, Escalab 250xi, Thermofisher, Waltham, MA, USA). Information on the specific surface area and pore size distribution of the powder samples was analyzed by N_2_ adsorption and desorption experiments at 77 K with a physical adsorption instrument (Autosorb iQ) manufactured by Quantachrome Instruments (Boynton Beach, FL, USA).

### 2.3. Electrochemical Performance Tests

Electrochemical performance tests were conducted at room temperature with a three-electrode electrochemical cell attached to an electrochemical workstation (CHI660E, China). All samples were tested with an electrolyte of 6 M KOH with a platinum counter electrode and saturated glycerol reference electrode. The working electrodes were prepared by mixing the active substance, PTFE, and acetylene black in an 8:1:1 mass ratio and then pressing the mixture onto nickel foam at 8–10 MPa. Electrochemical impedance spectroscopy (EIS) data were measured in the frequency range of 0.01–100 KHz.

## 3. Results and Discussion

### 3.1. Material Characterization

**Figure 1:**
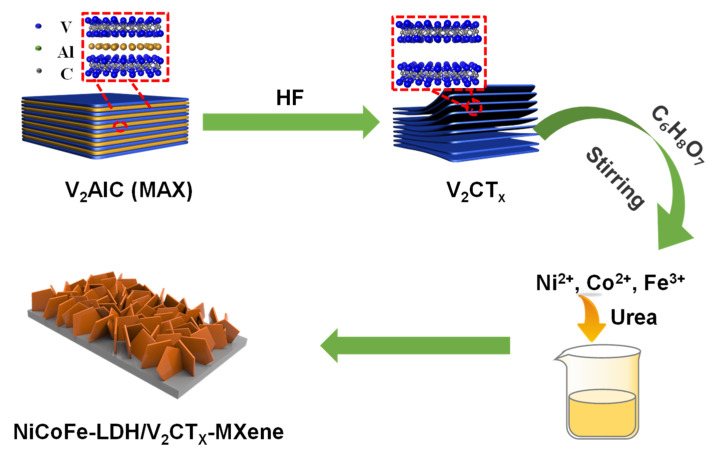
Schematic illustrating the preparation of NiCoFe–LDH/V_2_CT_x_–MXene.

Figure 1 illustrates the synthesis of three-dimensional interconnected network NiCoFe–LDH/V_2_CT_x_–MXene heterostructured composites. The V_2_CT_x_–MXene substrate material with an accordion-like structure was obtained by etching V_2_AlC with HF, followed by the addition of Ni(NO_3_)_2_·6H_2_O, Co(NO_3_)_2_·6H_2_O, Fe(NO_3_)_3_·9H_2_O, and urea, which provide Ni^2+^, Co^2+^, Fe^3+^, and OH^−^, respectively. This was accomplished by introducing the nanosheet network structure of NiCoFe–LDH onto the hydrothermal MXene surface.

**Figure 2:**
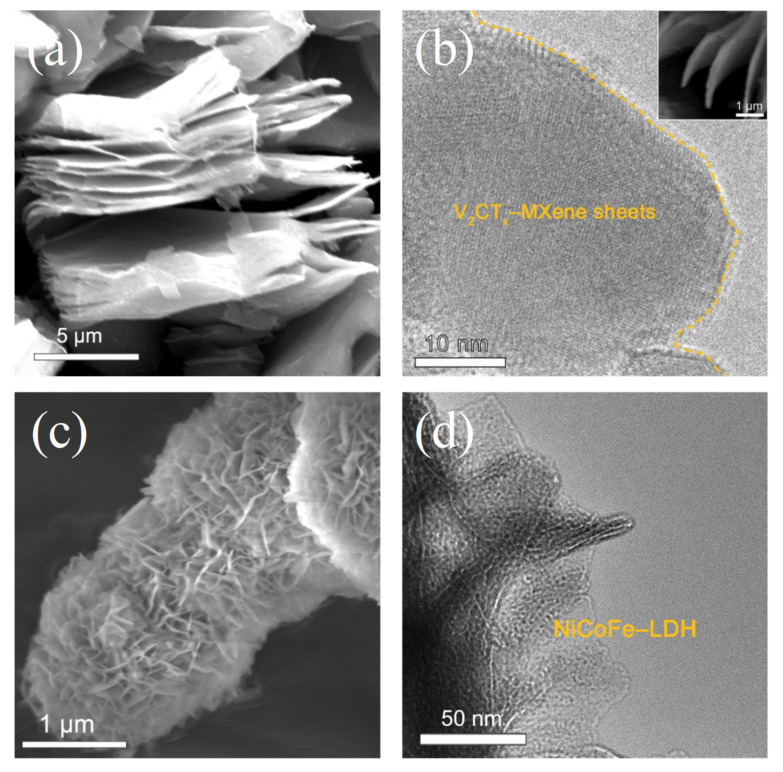
(**a**) Field emission SEM images of delaminated V_2_CT_x_–MXene sheets. (**b**) Field emission TEM images of few-layer V_2_CT_x_–MXene sheets. An SEM image is shown in the inset. (**c**) SEM and (**d**) TEM images of NiCoFe–LDH/V_2_CT_x_–MXene.

The morphology and composition of the samples were investigated using SEM and TEM. Figure 2a,b show the variation in layer spacing of V_2_CT_x_–MXene before and after the dispersion treatment. The layerless sheets obtained after intercalation and sonication are more favorable for the growth of Ni^2+^, Co^2+^, and Fe^3+^ metal ions. A SEM panoramic view shows the three-dimensional interconnected layered NiCoFe–LDH nanosheets on the layerless V_2_CT_x_–MXene nanosheets with uniform distribution (Figure 2c). TEM images further demonstrate the growth of several nanometer-thick arrays of NiCoFe–LDH nanosheets on the surface of the V_2_CT_x_–MXene substrate material, as shown in Figure 2d. This structure can significantly shorten the conversion and ion diffusion paths of the electrode material during redox reactions [19,20].

**Figure 3:**
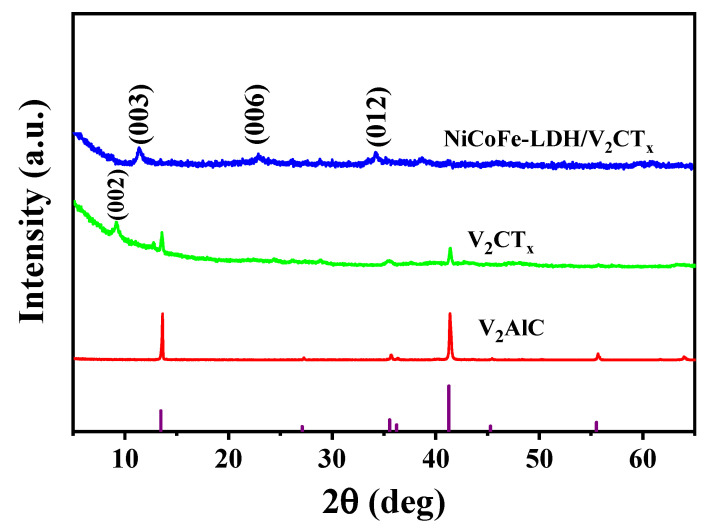
XRD pattern of NiCoFe–LDH/V_2_CT_x_.

The X-ray diffraction (XRD) patterns of V_2_AlC–MAX, V_2_CT_x_–MXene, and NiCoFe–LDH/V_2_CT_x_–MXene are shown in Figure 3. The V_2_AlC–MAX powder (PDF#29-0101) was etched for 95 h using HF, which successfully transformed it from the MAX phase to the MXene phase. The peaks at 2θ = 13.466° and 41.265° indicate the presence of some residual Al phase and the peak at 2θ = 9.09° belongs to the (002) crystal plane. The (002) peak of the V_2_CT_x_–MXene phase was not present for the NiCoFe–LDH/V_2_CT_x_–MXene material, which may be due to the coverage of the NiCoFe–LDH nanosheet array [19]. All the diffraction peaks can be indexed to JCPDS No. 38-0715 [21].

**Figure 4:**
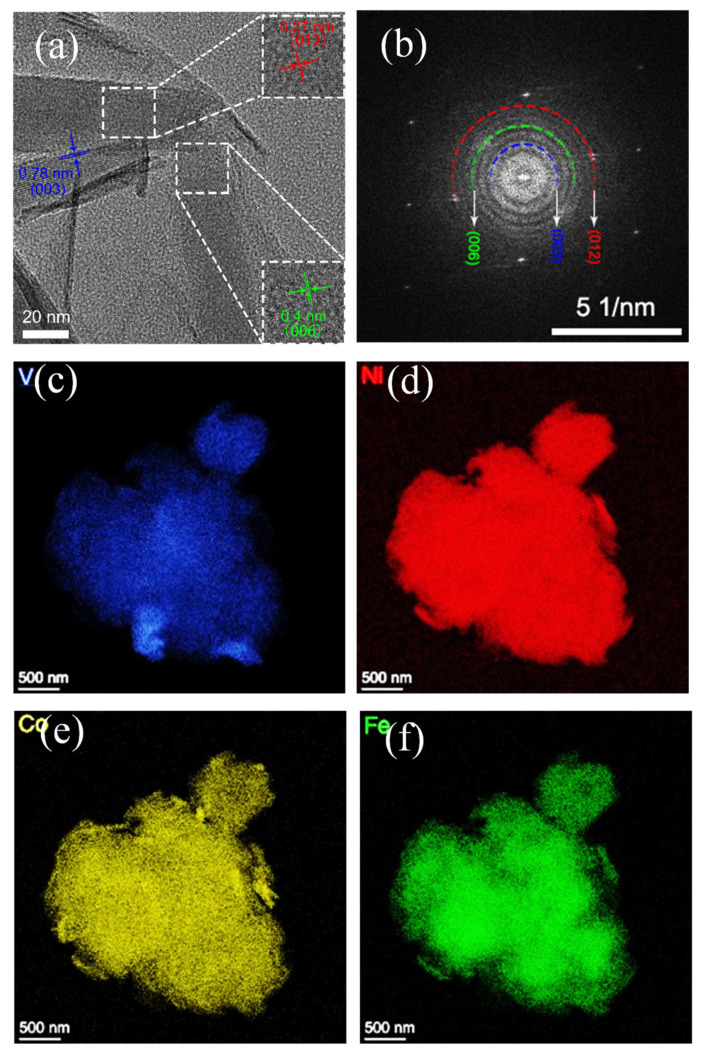
(**a**) HRTEM image; (**b**) SAED image; Elemental mapping of (**c**) V, (**d**) Ni, (**e**) Co, and (**f**) Fe in NiCoFe–LDH/V_2_CT_x_–MXene.

To further confirm the presence of the NiCoFe–LDH phase, HRTEM tests were conducted, as shown in Figure 4a. The crystal plane spacings at distances of 0.78, 0.4, and 0.26 nm correspond to the diffraction crystal planes of NiCoFe–LDH as (003), (006), and (012) crystal planes, respectively. Figure 4b shows the corresponding SAED patterns with three labeled concentric bright rings, which match the XRD lattice information at 11.34° (003), 22.73° (006), and 34.41° (012) (Figure 3), confirming the polycrystalline character of NiCoFe–LDH. The EDS mapping shows the uniform distribution of V, Ni, Co, and Fe on the surface of NiCoFe–LDH/V_2_CT_x_–MXene.

**Figure 5:**
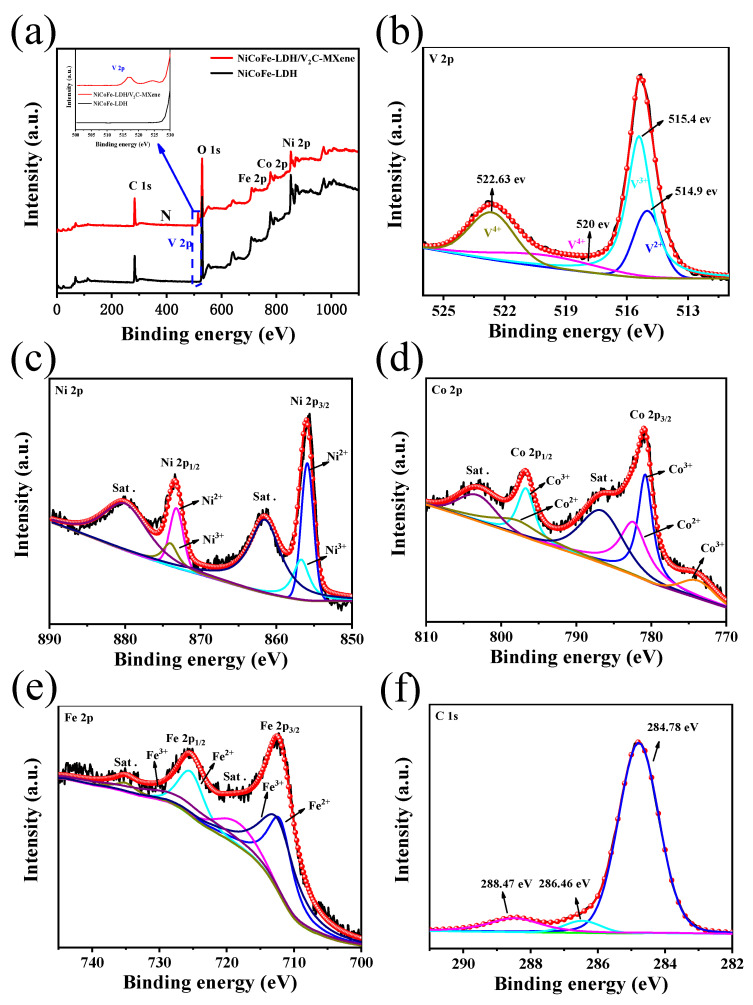
(**a**) XPS measurement scans of NiCoFe–LDH and NiCoFe–LDH/V_2_CT_x_–MXene. The insets show the enhanced XPS profiles in the range of 512–527 eV. The (**b**) V 2p, (**c**) Ni 2p, (**d**) Co 2p, (**e**) Fe 2p, and (**f**) C 1s XPS profiles of NiCoFe–LDH/V_2_CT_x_–MXene.

XPS was performed to better investigate the elemental types and chemical valence states of NiCoFe–LDH and NiCoFe–LDH/V_2_CT_x_–MXene, as shown in Figure 5a–f. The spectra show the elements V, Ni, Co, Fe, and C, which are consistent with the elemental mapping results. All elements were corrected for carbon at 284.8 eV. The binding energy at 514.9 eV in the high-resolution V 2p spectrum (Figure 5b) indicates the presence of V_2_AlC–MAX, with V^3+^, V^4+^, and V^4+^ represented at binding energies of 515.4, 520, and 522.6 eV, respectively [22]. The Ni 2p spectrum has two spin-orbit double peaks in 2p_1/2_ and 2p_3/2_, with binding energies of 873.4 and 855.6 eV, respectively (Figure 5c). The binding energies at 855.8, 856.6, 873.1, and 874.3 eV demonstrate the coexistence of Ni^2+^ and Ni^3+^ in the samples [23]. In the Co 2p spectrum (Figure 5d), two peaks, namely 2p_1/2_ and 2p_3/2_, can be observed at binding energies of 797.1 and 780.9 eV, respectively. The peaks at 780.7 and 796.6 eV indicate the presence of Co^3+^, while the peaks at 782.3 and 797.8 eV indicate the presence of Co^2+^ [24]. This proves that Co^2+^ and Co^3+^ is present in NiCoFe–LDH/V_2_CT_x_–MXene. Two spin-orbit double peaks at binding energies of 712.1 and 725.4 eV for 2p_1/2_ and 2p_3/2_, respectively, are visible in the Fe 2p spectrum shown in Figure 5e. The asymmetry of the spectral peak positions reveals the presence of two valence states of Fe^2+^ and Fe^3+^ [25]. In the C 1s spectrum shown in Figure 5f, three peaks corresponding to binding energies of 284.78, 286.46, and 288.47 eV are visible. The highest intensity of these peaks is at a binding energy of 284.78 eV, which is for the sp^2^-hybridized graphitic carbon [26]. The two peaks at 286.46 and 288.47 eV correspond to defects and C=O, respectively [27,28].

**Figure 6:**
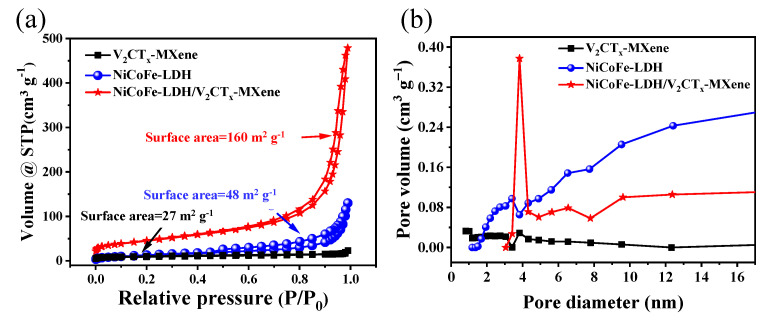
(**a**) N_2_ adsorption–desorption isotherms. (**b**) Pore size distribution of V_2_CT_x_–MXene, NiCoFe–LDH, and NiCoFe–LDH/V_2_CT_x_–MXene.

The specific surface area and pore size distribution data of the samples were obtained by analyzing the N_2_ adsorption–desorption isotherms and pore size distribution plots of the samples (Figure 6a,b). As shown in Figure 6a, the NiCoFe–LDH/V_2_CT_x_–MXene sample shows a typical IV isotherm with a hysteresis line ranging from approximately 0.6–1.0 P/P0, which indicates the presence of a large number of mesopores and micropores in the sample. The BET equation was used to calculate the specific surface area of NiCoFe–LDH/V_2_CT_x_–MXene sample (160 m^2^ g^−1^), which was much higher than that of NiCoFe–LDH (48 m^2^ g^−1^) and V_2_CT_x_–MXene (27 m^2^ g^−1^). This implies that V_2_CT_x_–MXene can inhibit the aggregation of NiCoFe–LDH nanosheets and uniformly distribute on the surface of the V_2_CT_x_–MXene substrate material, thus increasing the specific surface area of the material. These results prove the mesoporous nature of NiCoFe–LDH/V_2_CT_x_–MXene materials. The high specific surface area can provide more sites with the same mass that can create more favorable conditions for electrolyte-contacting electrode materials, which can effectively promote the charge transport of NiCoFe–LDH/V_2_CT_x_–MXene. The three-dimensional interconnected porous network microstructure can accelerate ion diffusion while increasing the specific surface area, which is more conducive to the material achieving rapid charge and discharge.

### 3.2. Electrochemical Performance

**Figure 7:**
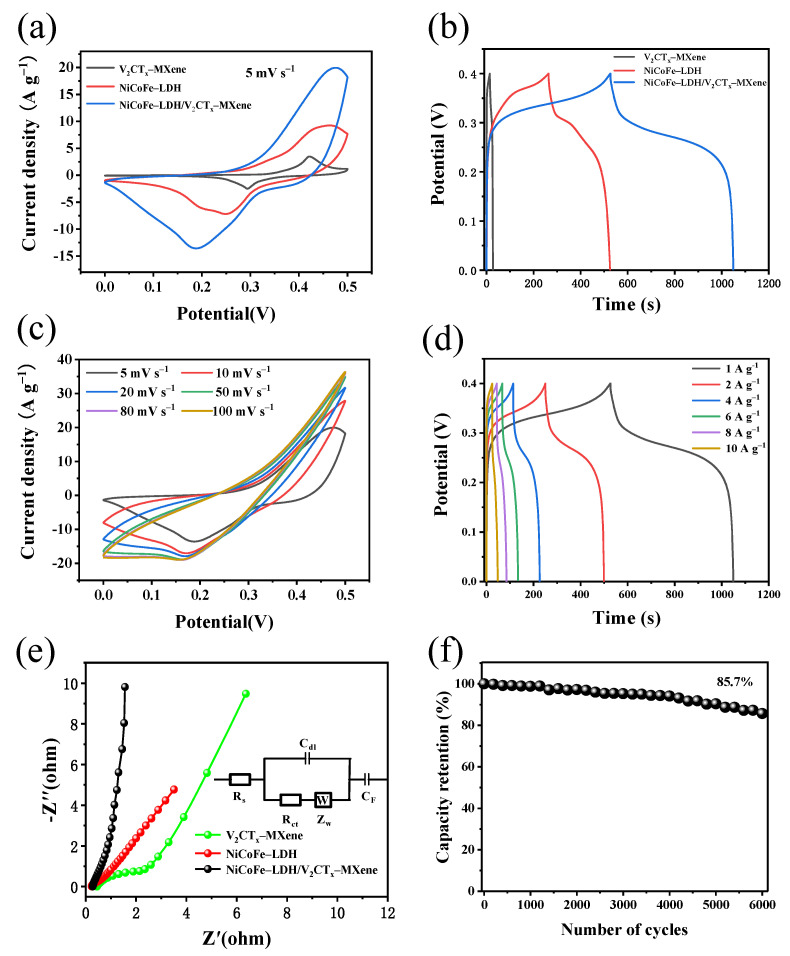
Comparison of the electrochemical performance of V_2_CT_x_–MXene, NiCoFe–LDH, and NiCoFe–LDH/V_2_CT_x_–MXene electrodes: (**a**) CV curve at 5 mV s^−1^; (**b**) GCD curve at 1 A g^−1^; NiCoFe–LDH/V_2_CT_x_–MXene; (**c**) CV curve; (**d**) GCD curve; (**e**) Nyquist plot (the inserted is the equivalent circuit); and (**f**) cycling stability at 10 A g^−1^.

CV tests were conducted on V_2_CT_x_–MXene, NiCoFe–LDH, and NiCoFe–LDH/V_2_CT_x_–MXene at a scan rate of 5 mV s^−1^ in a voltage window of 0–0.5 V. Figure 7a shows that all three samples have a pair of distinct redox peaks that exhibit a clear potential separation, which is clear pseudocapacitive behavior [29,30,31]. The area enclosed by the NiCoFe–LDH/V_2_CT_x_–MXene CV curve is larger than that of the other two samples, indicating that its specific capacitance is also the largest. Figure 7b shows the GCD curve at a current density of 1 A g^−1^. NiCoFe–LDH/V_2_CT_x_–MXene has the longest discharge time, echoing the conclusion of the CV curve. Thus, it was selected for the study. Figure 7c shows the CV curve of this sample. The peak position shifts with an increase in sweep speed because of the polarization effect at high sweep speeds. The reaction that mainly occurs on the electrode surface can be expressed by the following equations [32]:

(1) $$\mathrm{Co}(\mathrm{OH}{)}_{2}+{\mathrm{OH}}^{-}\;⇆\mathrm{Co}\mathrm{OOH}+H_{2}O+e^{-}$$

(2) $$\mathrm{CoOOH}+{\mathrm{OH}}^{-}\;⇆\mathrm{Co}O_{2}+H_{2}O+e^{-}$$

(3) $$\mathrm{Ni}(\mathrm{OH}{)}_{2}+{\mathrm{OH}}^{-}\;⇆\mathrm{Ni}\mathrm{OOH}+H_{2}O+e^{-}$$

The corresponding GCD curves are shown in Figure 7d and correspond to specific capacitances of 1305, 1245, 1120, 1005, 840, and 605 F g^−1^ for current densities of 1, 2, 4, 6, 8, and 10 A g^−1^, respectively.

The EIS was obtained in the range from 0.01 Hz to 0.1 MHz, as shown in Figure 7e. It can be seen that V_2_CT_x_–MXene has the smallest semicircle radius in the high frequency region and the largest slope of the straight line in the low-frequency region, indicating small ohms and fast electrolyte diffusion. After compounding with NiCoFe–LDH, the resistance to charge transfer and substance transfer increased. The charge transfer resistance (Rct) of NiCoFe–LDH/V_2_CT_x_–MXene (0.42 Ω) was significantly lower than that of NiCoFe–LDH (1.21 Ω), and V_2_CT_x_–MXene (2.16 Ω). This indicates that the presence of NiCoFe–LDH and V_2_CT_x_–MXene contributed to the resistance reduction and improved the electrode materials charge transfer rates.

Further, the NiCoFe–LDH/V_2_CT_x_–MXene electrode was charged and discharged for 6000 times at a current density of 10 A g^−1^ and the capacitance could still be maintained at 85.7% of the initial performance, indicating the excellent stability of the prepared electrode material. A comparison of the electrochemical properties of NiCoFe–LDH/V_2_CT_x_–MXene with the seven groups of LDH-based composites containing conductive matrices cited in Table 1 shows that the specific capacitance and stability of NiCoFe–LDH/V_2_CT_x_–MXene are better than those of other composites, indicating that it is more suitable as an electrode material compared to other composites and more suitable for applications in high-performance super electrical components.

**Figure 8:**
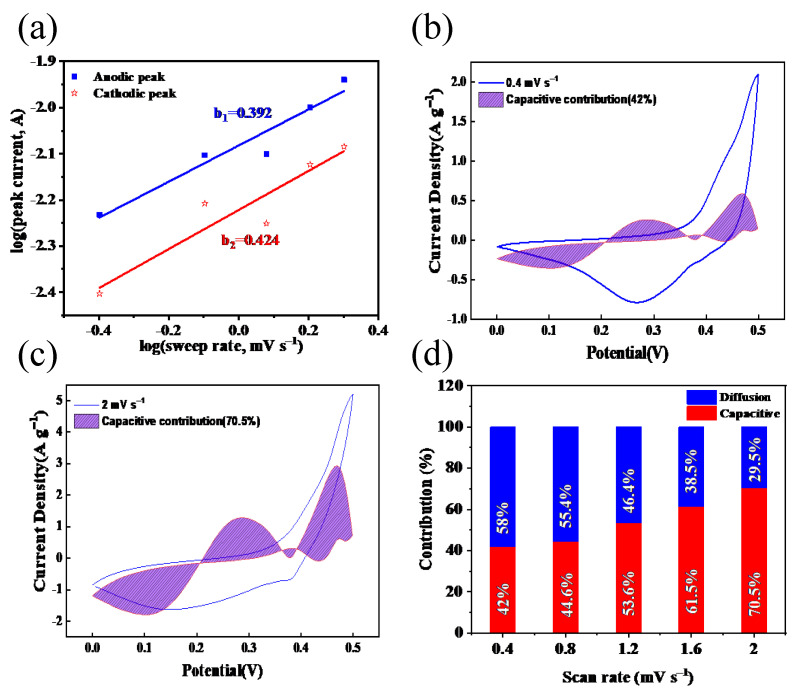
(**a**) Relationship between peak current and scan rate. Capacitance ratio fitting and CV curves of NiCoFe–LDH/V_2_CT_x_–MXene composites at (**b**) 0.4 and (**c**) 2 mV s^−1^. (**d**) Normalized contribution of the capacitance and diffusion control capacitance at different scan rates.

To understand the electrochemical kinetic process of energy storage in NiCoFe–LDH/ V_2_CT_x_–Mxene electrodes, CV curves were tested at scan rates of 0.4–2 mV s^−1^, as shown in Figure 8b,c. The connection between the peak current and the scan rate can be explained using the following equation [39,40,41]:

i = a *v*^b^, (4)

where *i* is the peak current, *v* is the scan rate, and *a* and *b* are constants. As shown in Figure 8a, the oxidation and reduction peaks of the NiCoFe–LDH/V_2_CT_x_–MXene electrode correspond to b values of 0.392 and 0.424. These data indicate that the NiCoFe–LDH/V_2_CT_x_–MXene electrode kinetics mainly follow a diffusion process. To further understand the contribution of the diffusion and surface processes to the capacity at different scan rates, a unified planning equation for a CV dynamics analysis was used [42,43].

Figure 8b,c show the CV curves at scan rates of 0.4–2 mV s^−1^ and the curves they are fitted to. Figure 8d shows the capacitance contributions of 42%, 44.6%, 53.6%, 61.5%, and 70.5% for sweep rates of 0.4, 0.8, 1.2, 1.6, and 2 mV s^−1^, respectively, and the capacitance contributions show a positive correlation with the sweep rate. At low sweep rates, the diffusion process plays a major role, while at high sweep rates, the surface capacitance plays a larger role owing to the diffusion delay at high sweep rates [44,45]. This result indicates the fast energy storage mechanism of the surface capacitance of NiCoFe–LDH/V_2_CT_x_–MXene.

To demonstrate the potential application of NiCoFe–LDH/V_2_CT_x_–MXene, a NiCoFe–LDH/V_2_CT_x_–MXene//AC double electrode period was assembled using NiCoFe–LDH/V_2_CT_x_–MXene as the positive electrode and AC as the negative electrode. In this case, the mass ratio of positive and negative electrodes was calculated as 0.383 according to the following equation.

(5) $$\frac{m_{+}}{m_{-}}=\frac{C_{-}\times \Delta V_{-}}{C_{+}\times \Delta V_{+}}$$

**Figure 9:**
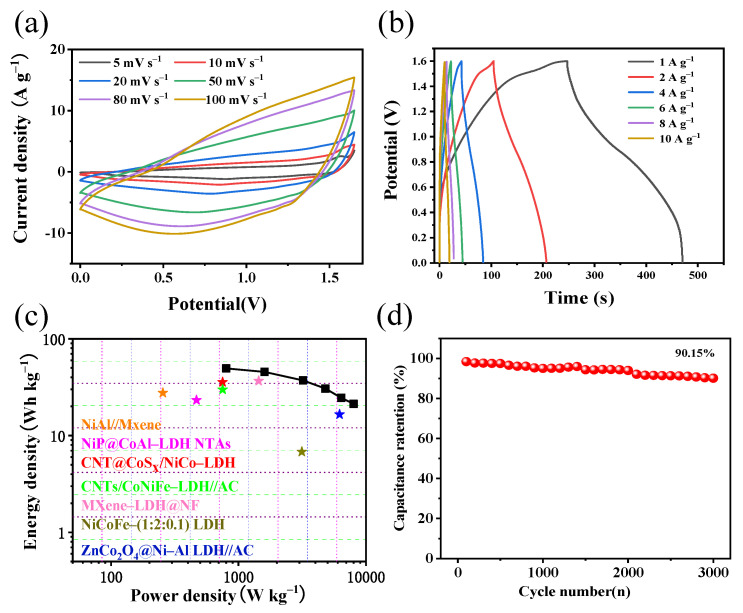
An asymmetric supercapacitor based on NiCoFe–LDH/V_2_CT_x_–MXene//AC: (**a**) CV curve; (**b**) GCD curve; (**c**) Ragone plot; and (**d**) cycling performance at 10 A g^−1^.

The CV curves of NiCoFe–LDH/V_2_CT_x_–MXene//AC were studied in a voltage window of 0–1.6 V (Figure 9). The profile of the curves remained the same as the sweep rate gradually increased from the low sweep rate, revealing that NiCoFe–LDH/V_2_CT_x_–MXene//AC can still maintain its original performance under fast charging and discharging conditions [46]. Figure 9b shows the GCD curve of NiCoFe–LDH/V_2_CT_x_–MXene//AC with a specific capacitance of 139 F g^−1^ at 1 A g^−1^. The small charging and discharging plateau and the shape of the triangular curve indicate that both positive and negative electrodes contribute to the specific capacitance of NiCoFe–LDH/V_2_CT_x_–MXene//AC.

Figure 9c shows the Ragone plot of NiCoFe–LDH/V_2_CT_x_–MXene//AC, which corresponds to 49.5 Wh kg^−1^ at 800 W kg^−1^ and 8000 W kg^−1^ at 21.3 Wh kg^−1^ for this double electrode. The performance always manifests competitively in practical utilization compared with previous reports, such as NiAl//MXene (27.6 Wh kg^−1^ at 255 W kg^−1^) [37], NiP@CoAl–LDH NTAs (23.27 Wh kg^−1^ at 468 W kg^−1^) [47], CNT@Co$S_{x}$/NiCo–LDH (35.64 Wh kg^−1^ at 750 W kg^−1^) [48], CNTs/CoNiFe–LDH//AC (29.9 Wh kg^−1^ at 750.5 W kg^−1^) [49], MXene–LDH@NF (36.7 Wh kg^−1^ at 1440 W kg^−1^) [50], NiCoFe-(1:2:0.1) LDH (6.8 Wh kg^−1^ at 3139.2 W kg^−1^) [51], and ZnC$o_{2}O_{4}$@NiAl LDH//AC (16.53 Wh kg^−1^ at 6200 W kg^−1^) [52]. Figure 9d shows the NiCoFe–LDH/V_2_CT_x_–MXene//AC electrode. The charge/discharge stability at 10 A g^−1^ shows that the capacitance performance of the double electrode remains at 85.3% after 10,000 cycles, which is comparable to that of the triple electrode. This indicates that it still has excellent application value after being assembled into a device.

## 4. Conclusions

Herein, a three-dimensional interconnected porous network microstructure of NiCoFe–LDH nanosheet arrays grown on V_2_CT_x_–MXene sheet layers was fabricated by HF etching and a hydrothermal method to composite V_2_CT_x_–MXene with LDH, which eased the stacking of NiCoFe–LDH nanosheets and maximized the specific surface area of the composite. The unique structure imparted NiCoFe–LDH with a large active surface and a continuous conductive pathway. NiCoFe–LDH/V_2_CT_x_–MXene exhibited a specific capacitance of 1305 F g^−1^ at 1 A g^−1^, and the performance of the prepared electrode material could be maintained at 85.7% after 6000 charge/discharge tests at high currents. When assembled into a NiCoFe–LDH/V_2_CT_x_–MXene//AC device with AC as the negative electrode and the prepared electrode material as the positive electrode, its specific capacitance was 139 F g^−1^ at 1 A g^−1^, and the charge/discharge curve had a distinct charge/discharge plateau, corresponding to 49.5 Wh kg^−1^ at 800 W kg^−1^. This indicated that the electrode could achieve a fast charge/discharge process.

## Figures and Tables

**Table 1:** Comparison of the electrochemical performance of NiCoFe–LDH/V_2_CT_x_–MXene with LDH-based composites containing other conductive substrates.

Composites	Specific Capacitances at Different Current Densities (F g^−1^)			Current Density (A g^−1^), Number of Cycles	Capacitance Retention (%)	Reference
	1 A g^−1^	2 A g^−1^	10 A g^−1^			
Ti_3_C_2_T_10_/NiCo–LDH	-	730	580	4, 2000	81	[15]
NiMn–LDH/V_2_CT_x_–Mxene	1005	836	570	-	-	[33]
M30/LDH	1061	-	-	4, 4000	70	[34]
NiCoAl–LDH–MWCNT	1035	974	597	6, 1000	83	[35]
RGO/CoAl–LDH	825	752	-	4, 4000	89.3	[36]
NiAl–LDH/Mxene	1600	1453	-	10, 3000	78	[37]
NiCo–LDH/MLG	1212.75	1163.5	-	6, 3000	80.5	[38]
Present study	1305	1245	605	10, 6000	85.7	-

## Data Availability

Data will be made available on request.
